# Structural control of a novel hierarchical porous carbon material and its adsorption properties

**DOI:** 10.1038/s41598-022-06781-9

**Published:** 2022-02-24

**Authors:** Li-Feng Cai, Jie-Ming Zhan, Jie Liang, Lei Yang, Jie Yin

**Affiliations:** 1 College of Environmental and Biological Engineering, Fujian Provincial Key Laboratory of Ecology-Toxicological Effects and Control for Emerging Contaminants, Putian University, Putian, 351100 Fujian People’s Republic of China; 2 Department of Materials Science, Fudan University, Shanghai, 200433 People’s Republic of China

**Keywords:** Pollution remediation, Organic-inorganic nanostructures, Structural properties, Synthesis and processing

## Abstract

Novel hierarchical porous carbon materials (HPCs) were fabricated via a reactive template-induced in situ hypercrosslinking procedure. The effects of carbonization conditions on the microstructure and morphology of HPCs were investigated, and the adsorption of methylene blue (MB) on HPCs was explored. The as-prepared HPCs has a hierarchical micro-, meso- and macropore structure, which results from the overlap of hollow nanospheres possessing microporous shells and macroporous cavities. The carbonization temperature, carbonization time and carbonization heating rate played important roles in tailoring the nanostructures of HPCs. The BET specific surface area and micropore specific surface area can reach 2388 m^2^ g^−1^ and 1892 m^2^ g^−1^, respectively. Benefitting from the well-developed pore structure, the MB removal efficiency can exceed 99% under optimized conditions. The adsorption kinetics and thermodynamics can be well described by a pseudo-second-order model and Langmuir model, respectively. Furthermore, such adsorption was characterized by a spontaneous endothermic process.

## Introduction

Organic porous materials are superior in considerable specific surface area (SSA) and stable chemical structure and are widely applied in the fields of adsorption, phase separation, catalysis and energy storeage^1–5^. For organic porous materials, research on porous structures is gradually moving in two directions^6^. One is to conduct in-depth research on the existing porous structure to further improve its performance^7^. For instance, the pore size distribution was adjusted for a microporous material that adsorbs carbon dioxide, as the number and size of the microporous structure determine the amount of carbon dioxide adsorption, and a matched porous structure can exhibit the maximum adsorption capacity for gas or small molecules under the same conditions^8,9^. The other is to combine various pore structures through chemical reactions and to make full use of different pore structures. The increasing demand for organic porous materials in emerging fields tends to require materials with various pore structures.

Hierarchically porous carbon materials (HPCs) with micropores, mesopores and macropores are widely used in emerging fields due to their diverse pore structures with high SSA and excellent chemical stability^10,11^. However, the traditional synthesis method of HPC is still complicated, leading to an uncertain porous structure and even making the pores easily collapse. It is urgent to find a proper synthesis method. The preparation methods of hierarchical porous carbon materials can be divided into two methods according to different synthesis methods, where one is the hard template method and the other is the soft template method^12,13^. The hard template needs to synthesize a template with a predetermined structure, followed by a series of postprocessing steps (such as carbonization). Then, a hierarchical porous carbon material can be prepared after washing the template. However, the straits of the hard template method are as follows: (1) The preprocessing of the template is difficult. For example, pretreatment of the silica template must achieve a certain grafting rate of the active groups to ensure the success of the subsequent cross-linking reaction. (2) The subsequent postprocessing is also complicated. The selection of a carbon source and proper reaction conditions can make HPC an excellent pore structure^14^.

**Figure 1:**
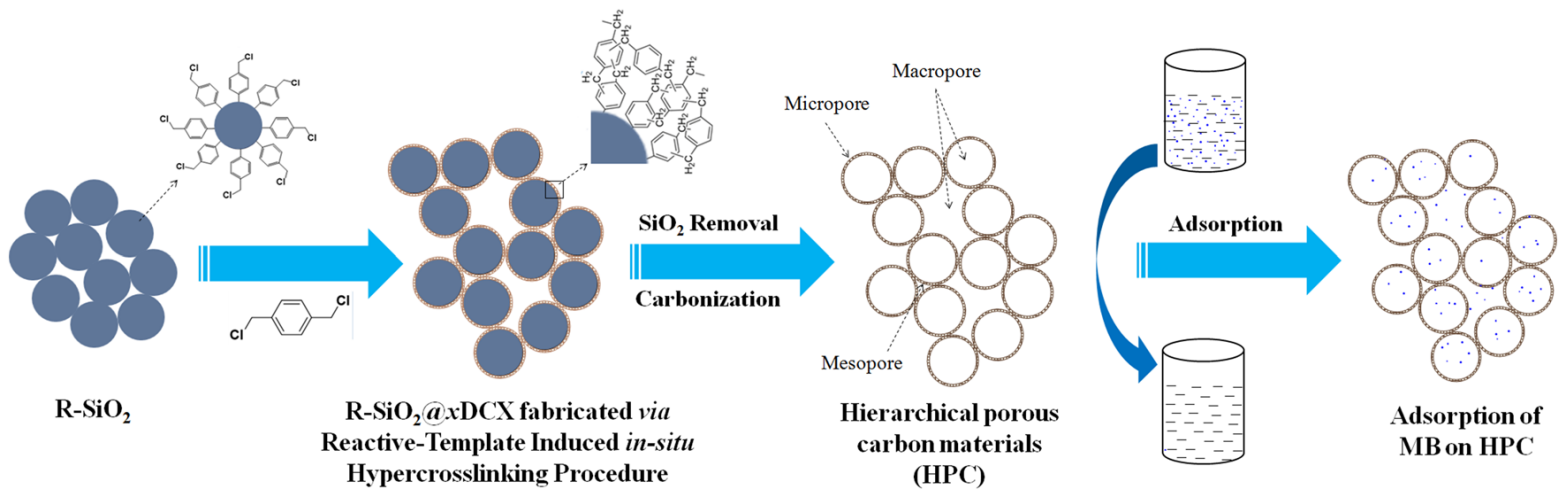
Schematic of novel hierarchical porous carbon material (HPC) and methylene blue (MB) adsorption.

Herein, a "reactive template-induced in situ hypercrosslinking method" was successfully developed for preparing hierarchical porous polymers (HPPs) and hierarchical porous carbon materials (HPCs) using functionalized SiO_2_ nanospheres as the template (R-SiO_2_) and 1,4-p-dichlorobenzyl (DCX) as the self-crosslinking functional monomer^15^. The HPPs and HPCs prepared from this method contain a unique porous structure of micropores-mesopores-macropores in a hierarchical distribution: the microporous shell and hollow nanospheres were cross-linked and stacked with each other (Fig. 1). The abundant micro/meso/macropores are closely connected, showing a synergistic effect, which improves the properties of the materials. The effect of hypercrosslinking time on the nanostructure of HPCs was studied in detail, which revealed the correlation between preparation conditions and the structural morphology of HPCs. In addition, the adsorption performance of HPCs toward methylene blue (MB) solution was further discussed, which provided a theoretical basis for the controllable preparation of HPCs for its application in the field of dye wastewater treatment.

## Experimental section

### Preparing for reactive SiO_2_ nanospheres (R-SiO_2_) and R-SiO_2_@xDCX

SiO_2_ nanospheres with an average diameter of 150 nm were synthesized through the Stöber method. SiO_2_ nanospheres were preprocessed to obtain reactive SiO_2_ nanospheres (R-SiO_2_). SiO_2_ nanospheres (0.5 g) were added to 20 mL of anhydrous tetrahydrofuran and 1.5 g of trichloro[4-(chloromethyl)phenyl]silane to a mixture with drastic stirring. Then, 5.0 mL anhydrous tetrahydrofuran and 1.2 mL of triethylamine mixture were added under nitrogen and reacted in a nitrogen/air atmosphere for 8 and 18 h, respectively, and then reacted in air for 18 h. The resulting solid precipitate (named R-SiO_2_) was filtered and sequentially washed with ethanol and tetrahydrofuran and vacuum-dried at 60 °C overnight.

Generally, 0.5 g of R-SiO_2_ nanospheres and 1.6 g of anhydrous ferric chloride were dispersed uniformly in 10 mL dichloroethane. Then, the system was added dropwise into a solution of p-dichlorobenzene in dichloroethane at a constant speed under the protection of nitrogen after reaching 80 °C. It was conducted a hypercrosslinked reaction on the R-SiO_2_ surface by means of the Friedel–Crafts reaction. The hypercrosslinking time was continuously extended from 3 to 24 h. The as prepared material was named as R-SiO_2_@xDCX-X, where the X was the hypercrosslinking time of the reaction.

### Synthesis of hierarchical porous polymer (HPP) and hierarchical porous carbon material (HPCs)

R-SiO_2_@xDCX-X was etched with 10% HF for 24 h. The precipitate was filtered and washed with ammonia and pure water to remove the residual HF. Then, the resulting product was vacuum-dried at 60 °C overnight to obtain a HPP-X, where the X was corresponding to the hypercrosslinking time from 3 to 24 h. Specifically, the HPP-24 was carbonized at various temperatures extended from 700 to 1000 °C in a tube furnace under the protection of a N_2_ atmosphere with a flow rate of 80 mL min^−1^ to obtain a hierarchical porous carbon materials (HPCs).

### Characterization

Scanning electron microscopy (SEM) images were obtained on an S8010 instrument (Hitachi, Japan) at an acceleration voltage and current of 10 kV and 10 μA, respectively. The N_2_ adsorption–desorption isotherm of the sample was measured at 77 K by an ASAP 2460 adsorption instrument (Micromeritics, USA). The BET surface area (*S*_*BET*_) was analyzed by Brunauer–Emmett–Teller (BET) theory. The external surface area (*S*_*ext*_) was determined by t-plot theory, and then the micropore surface area (*S*_*mic*_) was obtained by subtracting the S_ext_ from the S_BET_. The total pore volume (*V*_*total*_) was calculated from the amount adsorbed at a relative pressure *P/P*_0_ of 0.997. The micropore volume (*V*_*mic*_) was determined by t-plot theory and BJH method, respectively. The pore size distribution was analyzed by original density functional theory (DFT) assuming slit pore with non-negative regularization and medium smoothing.

An inVia laser Raman spectrometer from Renishaw Company was used to measure the microcrystalline structure of the sample. The scanning range was from 800–2000 cm^−1^. The excitation wavelength was 514.5 nm with an exposure time of 30 s.

### The adsorption performance test

A certain amount of methylene blue (MB) solution was added to an Erlenmeyer flask, and then HPC was added. The system was kept in a water bath with magnetic field at room temperature. It was placed in a constant temperature water bath with magnetic stirring for adsorption experiments. The solution was filtered with a microporous membrane, and the concentration of the filtrate was further measured by a 722 visible spectrophotometer (Shanghai Precision Scientific Instrument Co., Ltd.). The MB adsorption rate (*R*) was calculated according to formula (1):

1 $$R = \frac{{C_{0} - C_{{\text{e}}} }}{{C_{0} }} \times 100{{\% }}$$

In formula (1): *C*_0_ is the concentration of MB solution before adsorption (mg L^−1^); *C*_*e*_ is the concentration of MB solution at adsorption equilibrium (mg L^−1^);

The adsorption capacity (*q*) was calculated according to formula (2):

2 $$q = \frac{{(C_{0} - C{}_{t}) \cdot V}}{m}$$

In formula (2), *C*_0_ is the concentration of MB solution before adsorption (mg L^−1^), *C*_*t*_ is the concentration of MB solution at time *t* (mg L^−1^), *V* is the volume of MB solution (L), and *m* is the mass of HPC (g).

### Characterization of the HPP-Xs

**Figure 2:**
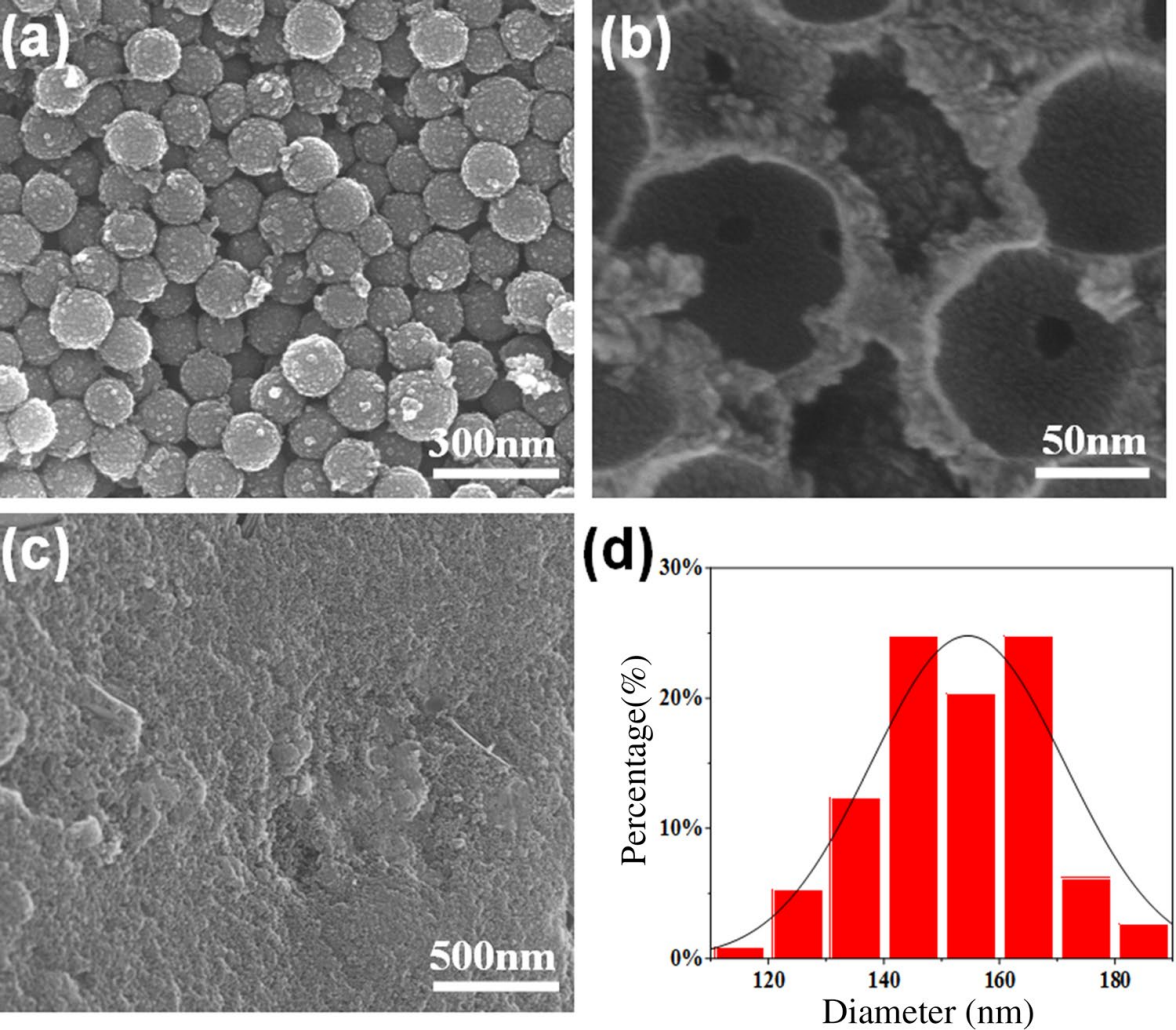
SEM graphs of (**a**) R-SiO_2_@xDCX-24 nanoarticles, (**b**) HPP-24, (**c**) SiO_2_@xDCX-24 nanoarticles, and (**d**) size distribution analysis of R-SiO_2_@xDCX-24 nanoarticles.

The successful preparation of R-SiO_2_ nanospheres was proven by a series of characterizations (Figs. S1–S3). The R-SiO_2_ nanospheres were further hypercrosslinked in situ by means of the Friedel–Crafts reaction. As shown in Fig. 2a, the surface of the R-SiO_2_@xDCX-24 nanospheres present a rough and uneven coating layer, proving the hypercrosslinked reaction on the R-SiO_2_ surface. The unmodified SiO_2_ was also used to react with DCX, while polymerization was carried out only between the DCX monomer. As shown in Fig. 2c, SiO_2_@xDCX-24 showed a flat and agglomerated surface, indicating that the cross-linking reaction did not occur. After removing the silica template (Fig. 2b), the internal cavity of the R-SiO_2_@xDCX-24 nanosphere maintains a complete spherical structure. The Fig. 2d shows that the average size of R-SiO_2_@xDCX-24 nanoarticles is 150 nm. In addition, a series of characterizations illustrate the successful preparation of R-SiO_2_@xDCX-24 (Figs. S4–S7).

**Figure 3:**
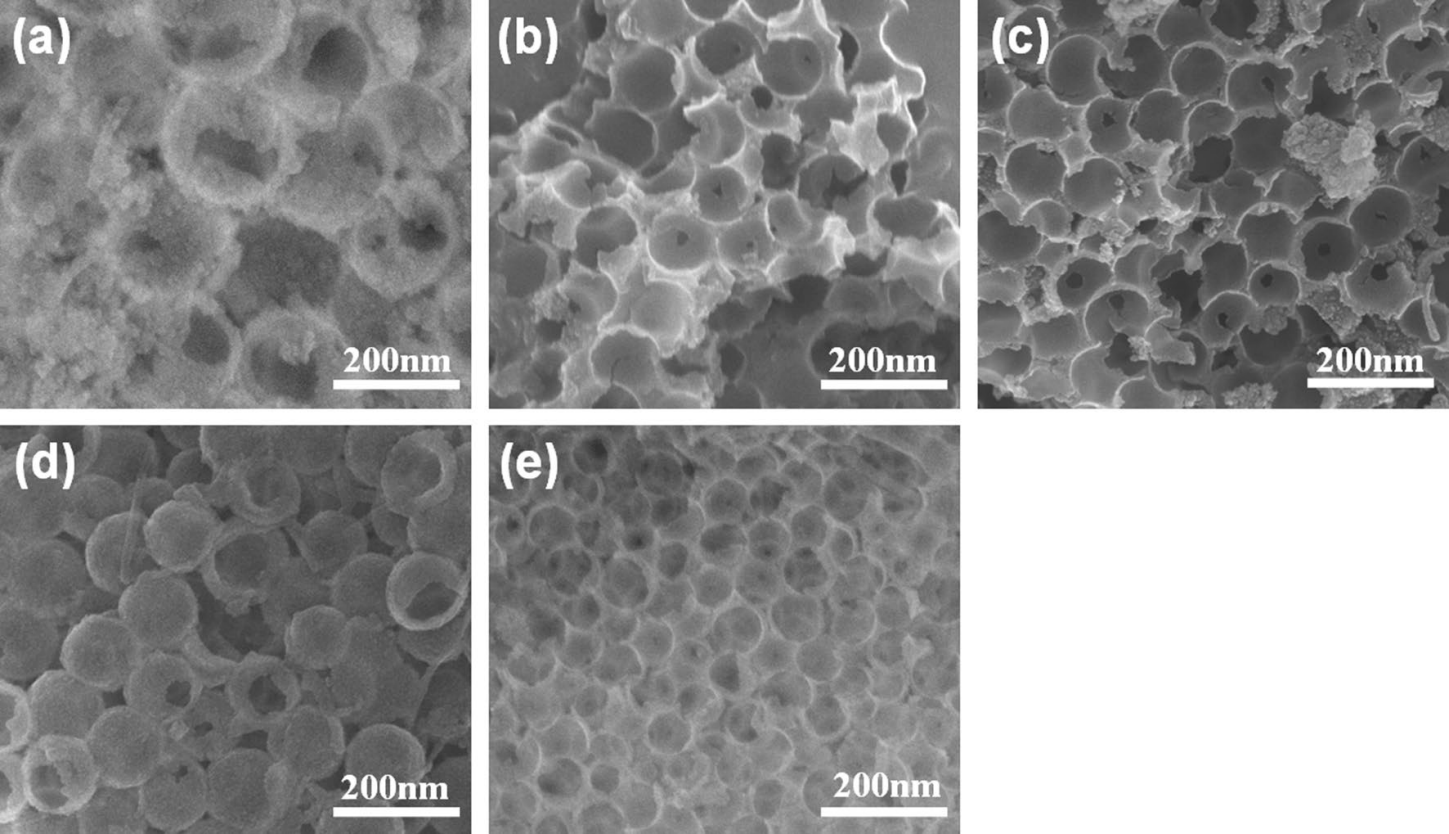
SEM graphs of HPP with various hypercrosslinking time (**a**) 3 h; (**b**) 6 h; (**c**) 12 h; (**d**) 18 h; (**e**) 24 h.

For carbon materials, the pore structure and element formation are mainly inherient from precursors^16–18^. Thus, the hypercrosslinking time was changed to explore the variation in the morphology and structure of HPP-Xs to precisely control the nanostructure of HPP-Xs. Figure 3 (SEM characterization) shows that the samples all contained structures with hollow and crossing spheres when the hypercrosslinking time was continuously extended from 3 to 24 h. As the hypercrosslinking time was just 3 h (Fig. 3a), the sample showed that a small part of the spherical shells were not connected with only a small part of the crosslinking hollow spheres, and the graft layer originally covered the spherical surface was almost peeled off, leaving a few residues. As the cross-linking time was extended to 6 h (Fig. 3b), the particle size of the sample tended to be uniform, while agglomeration still occurred, and hollow spheres were almost formed with a complete appearance. When the time reached 24 h (Fig. 3e), the hollow nanosphere shells were well stacked and regularly arranged together, forming a hollow hole-like structure with good morphology. The oligomer on the surface of the nanospheres gradually decreased with the extension of the hypercrosslinking time, which was caused by the gradual consumption of the polymerized part, leading to a more complete reaction^19^.

**Figure 4:**
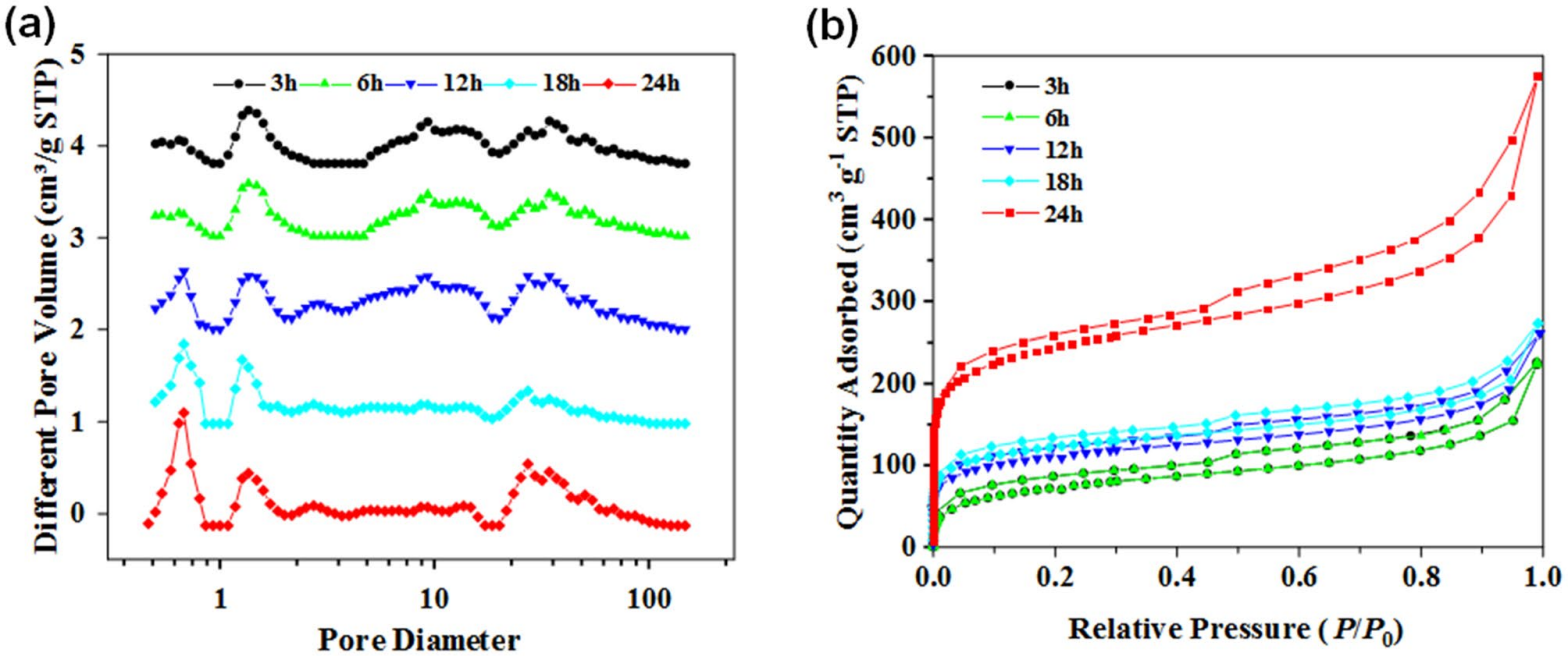
(**a**) DFT pore size distributions and (**b**) N_2_ adsorption desorption isotherms of HPP with different hypercrosslinking temperatures.

As shown in Fig. 4, all the samples presented a typical hierarchical porous structure that included micropores, mesopores and macropores^20,21^. The pore size distribution of HPP-Xs based on DFT calculations (Fig. 4a) showed peaks at 0.67 nm and 1.2 nm, at 27 nm and at 50–100 nm, corresponding to micropores, mesopores and macropores, respectively. The micropores were derived from the methylene bridge voids of the cross-linking between the nanospheres. As the Friedel–Crafts reaction was rapid, once the reaction occurred, it quickly polymerized to form a methylene cross-linked bridge to form a microporous structure. The changes in the hypercrosslinking reaction times deeply affected the crosslinking degree of the samples^22^.

Table 1 summarized the DTF calculation results based on the nitrogen absorption/desorption tests. As the reaction time was extended, the SSA and *V*_*mic*_ of the samples increased^23^. HPP-3 showed an SSA of 386 m^2^ g^−1^ and a *V*_*mic*_ of 0.04 cm^3^ g^−1^. As the crosslinking time of HPP-3 was shortened, the reaction could not proceed completely, leading to meso/macropores occluding the majority of the pore structure. With the extension of the cross-linking time, the SSA and the *V*_*mic*_ of HPP-6, HPP-12, HPP-18 and HPP-24 showed an increasing trend. The maximal SSA and *V*_*mic*_ were reached when the crosslinking time increased to 24 h, where *V*_*total*_ increased from 0.04 to 0.88 cm^3^ g^−1^ and the SSA increased from 386 m^2^ g^−1^ to more than double (889 m^2^ g^−1^). The results indicated that prolonging the super-crosslinking time was helpful to the Friedel–Crafts reaction, and the degree of cross-linking rose rapidly when the time reached 18 h. Comparing the value of *V*_*mic*_, it could be found that almost all the increase in the specific surface area came from the increase in the number of micropores. Since the microporous structure in the sample was mainly derived from the self-crosslinking reaction of the benzyl group between the benzene rings, the greater the degree of crosslinking between the spheres, the denser the cross-linked bridge structure, thus forming more micropores^24,25^. In conclusion, HPP-24 contained the highest SSAs and the largest number of micropores, which would be the best choice to be the precursor of HPCs.

**Table 1:** Pore structure parameters of HPP at various hypercrosslinking time.

Sample (h)	S_BET_ (m^2^ g^−1^)	S_mic_ (m^2^ g^−1^)	S_ext_ (m^2^ g^−1^)	V_mic_ (cm^3^ g^−1^)	V_ext_ (cm^3^ g^−1^)	V_total_ (cm^3^ g^−1^)
3	386	135	251	0.04	0.26	0.30
6	397	143	201	0.04	0.27	0.31
12	434	186	218	0.06	0.31	0.37
18	450	229	221	0.09	0.33	0.42
24	889	567	332	0.23	0.65	0.88

### The structure characterization of HPCs

**Figure 5:**
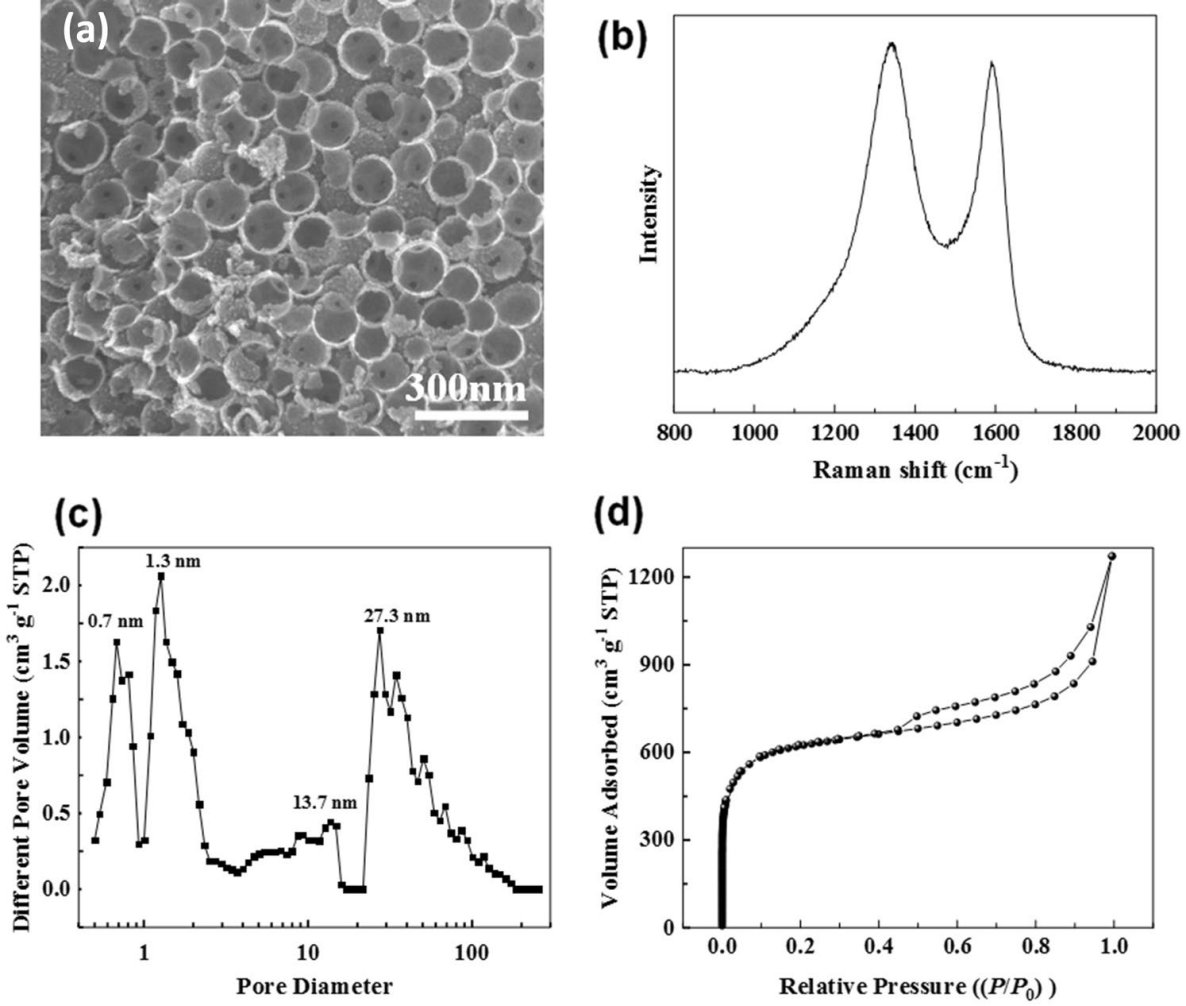
(**a**) SEM image, (**b**) Raman shift graph, (**c**) DFT pore size distribution and (**d**) N_2_ adsorption–desorption isotherm of HPC.

Figure 5a showed an SEM image of a typical HPC, displaying a three-dimensional nanonetwork structure with mesopores and macropores, which was formed by the stack of carbon nanospheres. The broken nanospheres possessed a hollow structure, indicating successful etching of the SiO_2_ template. Figure 5b show the Raman spectrum of HPC. The characteristic peak at 1345 cm^−1^ was the D peak, and the G peak was at 1590 cm^−1^, where the D peak corresponded to the vibration of carbon atoms and the G peak was the characterization of the graphitized structure, indicating that the carbon skeleton of HPC contained a graphite-like microcrystalline structure^26^. According to the following Tuinstra–Koenig formula (3):

3 $$L_{a} = { 4}.{35}I_{G} /I_{D}$$

In formula (3), *L*_*a*_ is length of graphite crystallite (nm), *I*_*G*_ and *I*_*D*_ are the fitted peak area of G peak and D peak, respectively. According to formula (3), the *L*_*a*_ of HPC is about 4.1 nm.

As shown in Fig. 5c, the pore size was distributed from the micropores (pore size below 2 nm) and the mesopores (pores from 2–50 nm) to the macropores (pores above 50 nm), indicating a hierarchical structure. Figure 5d illustrated the N_2_ adsorption–desorption isotherm of HPC. In the low-pressure region (*P*_0_*/P* < 0.1), the adsorption isotherm increased, indicating the microporous structure of HPC, while the isotherm showed a hysteresis loop area in the medium-pressure region, indicating the existence of mesopores; when the relative pressure was close to 1.0, the adsorption capacity increased significantly without the adsorption platform, showing the presence of macropores^27^. These conclusions were further proven by the pore size distribution curve, which was calculated from the DFT method.

**Figure 6:**
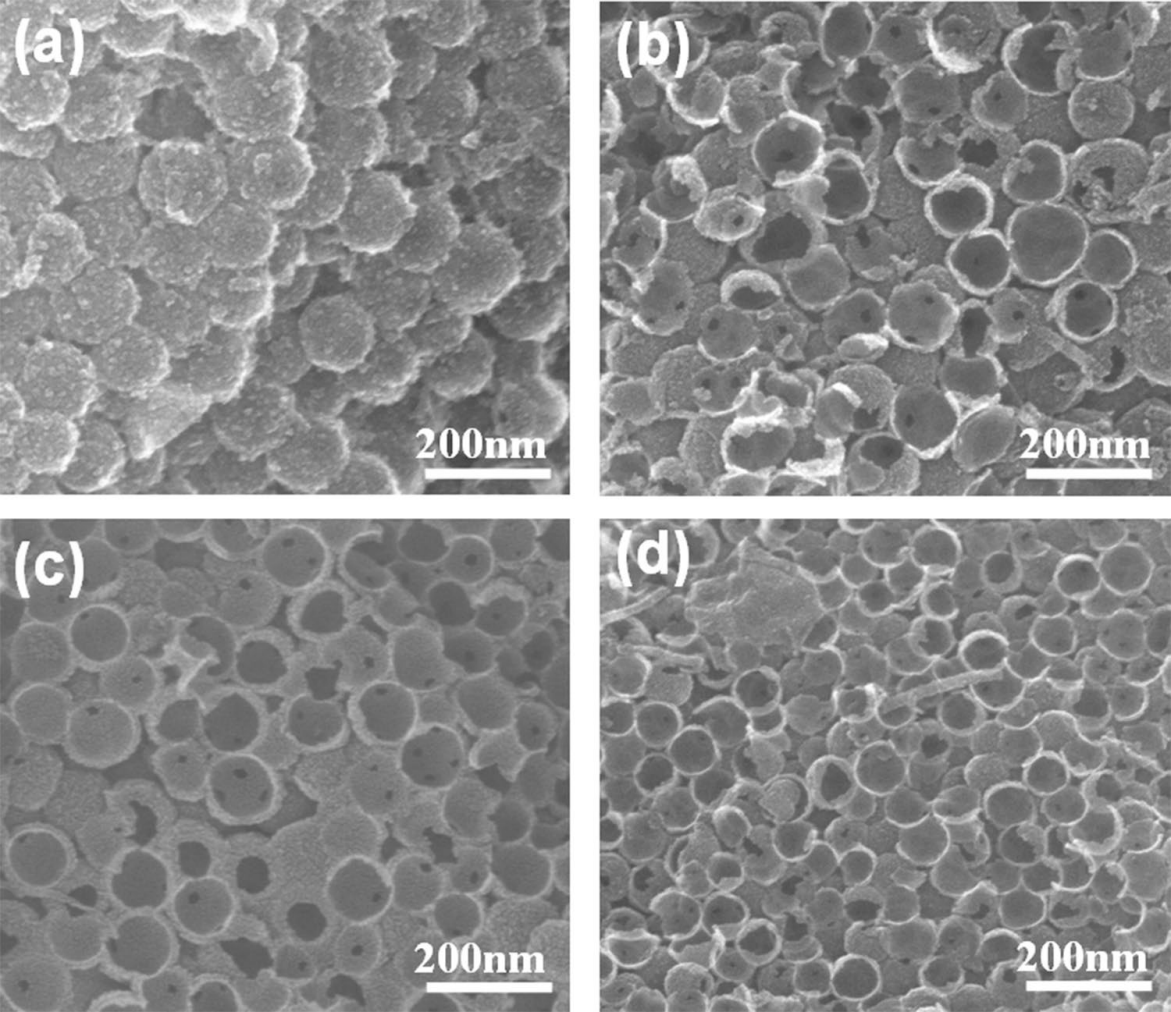
SEM images of HPC prepared from various carbonization temperatures: (**a**) 700 °C, (**b**) 800 °C, (**c**) 900 °C, (**d**) 1000 °C.

The influence of carbonization temperature on the HPCs’ nanostructure and SSA was further studied. Figure 6 showed the SEM image of HPCs prepared at different carbonization temperatures. This result showed that the special three-dimensional structure, which was formed by stacking hollow nanospheres, could be prepared at all carbonization temperature ranges, indicating that HPCs presents good thermal stability and framework strength. Table S1 summarizes the pore structure parameters of HPCs prepared under different carbonization conditions. The optimization conditions were a carbonization temperature of 1000 °C with a 3 h carbonization time and a carbonization heating rate of 5 °C min^−1^. The as-prepared HPCs presented a high SSA of 2388 m^2^ g^−1^ and an outstanding hierarchically porous structure.

### Adsorption performance of HPC

The results above showed that HPCs were microporous materials with abundant adsorption active sites. In addition, HPCs are hierarchically porous materials with connected pores, which could shorten the transmission distance between the pores, making HPCs a promising adsorbent with high-efficiency adsorption performance^28^. HPC-1000 with an SSA of 2388 m^2^ g^−1^ was selected as the adsorbent, and methylene blue (MB) was selected as the adsorbate to investigate the adsorption performance of HPCs. The mechanism of adsorption was discussed based on the model of adsorption kinetics, adsorption isotherm and adsorption thermodynamics.

**Figure 7:**
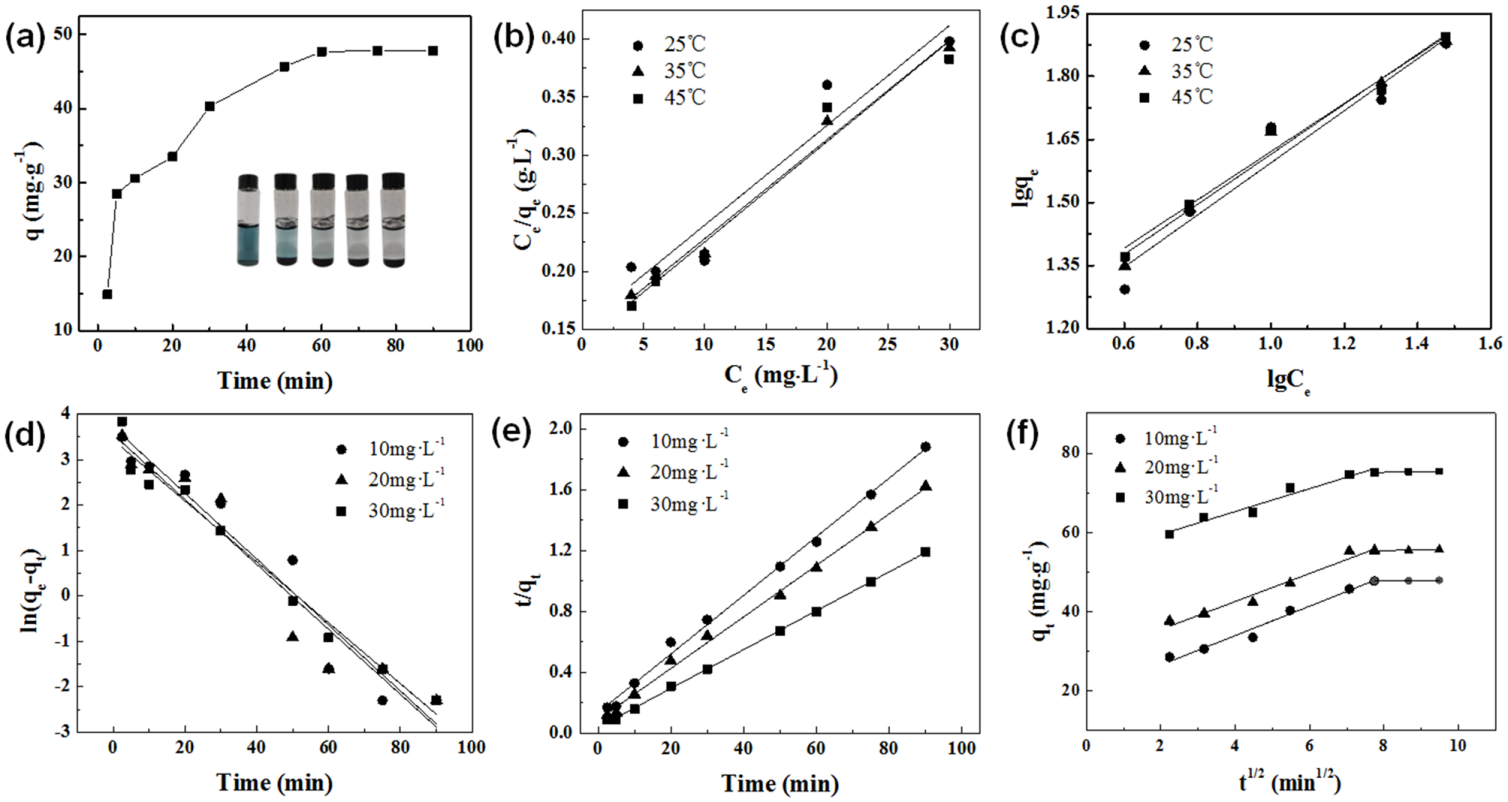
(**a**) Adsorption isotherms of MB on HPC, the inset shows digital photos of the absorption of MB by HPC in water, (**b**) Adsorption kinetics of MB on HPC treated by Langmuir isotherm, (**c**) Adsorption kinetics of MB on HPC treated by Freundlich isotherm, (**d**) Fitting results of pseudo-first-order kinetic model, (**e**) Fitting results of quasi-secondary kinetic model, (**f**) Fitting results of intraparticle diffusion model.

Figure 7 showed the adsorption kinetic curve of HPC-1000 in aqueous solution toward MB. When the adsorption time was 5 min, the adsorption capacity reached 28.5 mg g^−1^, and increased up to 47.7 mg g^−1^ at 60 min (the corresponding adsorption rate exceeded 99%), then did not change substantially as the adsorption time increased, indicating that the adsorption toward MB had reached equilibrium at this time. In the early stage of adsorption, the microporous structure of HPC-1000 had a large number of active sites, leading to a high initial rate of adsorption. As the adsorption time increased, the active sites inside HPC-1000 were filled with MB molecules, resulting in adsorption equilibrium after 60 min. Figure 7a illustrated a digital photo of HPC-1000 adsorbing toward MB in aqueous solution. As the time increased, the color of the MB solution became lighter and was completely colorless after 2 h, indicating the effective adsorption performance of HPC-1000 toward the MB solution. The maximum adsorption capacity of MB on various carbon adsorbents from literatures was listed in Table S4. According to the Table S4, HPC possesses a relatively larger specific surface area, which lead to an excellent adsorption performance (the maximum adsorption capacity can reach up to 658.2 mg g^−1^). Therefore, the as-prepared HPC exhibits a good application prospect in the field of dye wastewater treatment.

Figure 7 also showed the fitting results of the adsorption process using the pseudo-first-order kinetic model, pseudo-second-order kinetic model, and Weber–Morris intraparticle diffusion model^29^. Table 2 summarized the adsorption kinetic parameters. The correlation coefficient value (*R*^2^) of pseudo-second-order kinetics was higher with *R*^2^ values greater than 0.99. The equilibrium adsorption capacity (*q*_*e-exp*_) obtained in the experiment was very close to the theoretical adsorption capacity (*q*_*e-cal*_) calculated by the pseudo-second-order kinetic model, indicating that the pseudo-second-order kinetic model was more suitable for describing the adsorption process of HPC toward MB solution. The intraparticle diffusion model was used by the segmentation method to fit the adsorption kinetics data (Fig. 7f). As shown in Table 2, the linear correlation of the segmentation fitting was better (the *R*^2^ value can reach 0.9774), indicating the presence of intraparticle diffusion of MB adsorption on HPC. Considering that the dimension of MB is 1.43 nm × 0.61 nm × 0.40 nm and the above pore size distribution results, the diffusion could be divided into two stages, while the first stage was the rapid diffusion of MB in macropores and mesopores, and the second stage was the slow diffusion in micropores. Figure 7b,c showed the fitting results of the Langmuir model and Freundlich model^30^ of the thermodynamics of HPC adsorption toward MB solution. The fitting results were summarized in Table 3, where the linear correlation coefficients of the Langmuir model are all higher than those of the Freundlich model, indicating that the adsorption process was more according to the Langmuir isotherm model, which means the main monolayer adsorption of the adsorption process^31^. The study of the HPC adsorption thermodynamics toward MB solution was further explored. According to the influence of different temperatures on the adsorption in the range of 298–318 K, the adsorption thermodynamic constants can be calculated. As shown in Table 4, the Gibbs free energy ∆G value was less than zero at the experimental temperature. As the temperature rises, the absolute value of ∆G increases, indicating that HPC adsorption toward MB solution was a spontaneous behavior. The enthalpy change value (∆H) was more than 0, indicating that the adsorption of MB by HPC was an endothermic process. The entropy change value (ΔS) reflected the degree of disorder of the solid–liquid interface, and the value ΔS > 0 indicated that the adsorption process increased the degree of disorder of the molecular motion between the solid–liquid interface, indicating that HPC had a strong affinity toward MB solution^32^.

**Table 2:** Kinetic parameters at various MB concentrations.

Kinetic equation	*C*_0_ (mg L^−1^)	*q*_*e-exp*_ (mg g^−1^)	Kinetic parameters
Pseudo-first-order ln(*q*_*e*_-*q*_*t*_) = ln*q*_*e*_ − *K*_1_*t*	10	31.5	*K*_1_ = 0.07266, *q*_*e-cal*_ = 35.5, *R*^2^ = 0.9353
	20	32.3	*K*_1_ = 0.07159, *q*_*e-cal*_ = 35.1, *R*^2^ = 0.9285
	30	29.2	*K*_1_ = 0.06710, *q*_*e-cal*_ = 31.0, *R*^2^ = 0.9750
Pseudo-second-order *t/q*_*t*_ = 1/(*K*_2_*q*_*e*_^2^) + *t/q*_*e*_	10	48.1	*K*_2_ = 0.00279, *q*_*e-cal*_ = 51.8, *R*^2^ = 0.9956
	20	57.9	*K*_2_ = 0.00337, *q*_*e-cal*_ = 59.0, *R*^2^ = 0.9966
	30	77.8	*K*_2_ = 0.00439, *q*_*e-cal*_ = 78.2, *R*^2^ = 0.9991
Intraparticle diffusion *q*_*t*_ = *K*_*id*_* t*^1*/*2^ + *C*	10	–	*K*_*id1*_ = 3.71, *C*_1_ = 19.1, *R*^2^_1_ = 0.9774
			*K*_*id2*_ = 0.11, *C*_2_ = 46.8, * R*^2^_2_ = 0.9983
	20		*K*_*id1*_ = 3.53, *C*_1_ = 28.4, * R*^2^_1_ = 0.9619
			*K*_*id2*_ = 0.17, *C*_2_ = 54.1, * R*^2^_2_ = 0.9054
	30		*K*_*id1*_ = 2.89, *C*_1_ = 53.7, * R*^2^_1_ = 0.9505
			*K*_*id2*_ = 0.17, *C*_2_ = 73.8, * R*^2^_2_ = 0.9486

**Table 3:** Langmuir and Freundlich isothermal equation parameters.

Temperature (°C)	Langmuir			Frenndlich	
	*q*_m_ (mg g^−1^)	*K*_L_ (L mg^−1^)	*R*^2^	*K*_F_·(mg g^−1^)	*R*^2^
25	125.1	0.0522	0.94996	9.0346	0.92111
35	121.1	0.058	0.99316	10.8137	0.97651
45	123.2	0.0588	0.99726	10.305	0.96459

**Table 4:** Adsorption thermodynamic parameters.

Temperature(K)	Thermodynamic parameters		
	*∆G*·(kJ mol^−1^)	*∆H* (kJ mol^−1^)	*∆S* (J mol^−1^ K^−1^)
298	− 4.86		
308	− 5.34	14.910	41.947
318	− 5.70		

## Conclusion

In conclusion, this paper provides a novel "reactive template-induced in situ hypercrosslinking method" to synthesize hierarchical porous carbon materials (HPCs). The effect of carbonization conditions on the nanostructure and morphology of HPCs was deeply studied. The results show that the shell-macroporous hollow nanospheres were stacked on each other to form meso/macropores, forming a three-dimensional nanonetwork structure. The diameter of the hollow nanospheres was approximately 150 nm, presenting unique micropore–mesopore–macropore hierarchical distribution pore structure characteristics; by controlling the carbonization conditions, the pore structure of HPCs could be effectively customized. The adsorption rate of HPC toward methylene blue (MB) small molecules in aqueous solution could exceed 99%. In addition, the adsorption kinetics and thermodynamics toward MB were in line with the pseudosecondary adsorption kinetics model and Langmuir model. The adsorption process was homogeneous monolayer adsorption, and it was a spontaneous endothermic process.

## Supplementary Information

Supplementary Information.
