# Supplementary material for: Structural control of a novel hierarchical porous carbon material and its adsorption properties
Source: Sci Rep. 2022 Feb 24;12:3118. doi: 10.1038/s41598-022-06781-9 (PMC8873302; doi:10.1038/s41598-022-06781-9)
Supplement: Supplementary file 1 — Supplementary Information. [file 41598_2022_6781_MOESM1_ESM.docx]

**Structural control of a novel hierarchical porous carbon material and its adsorption properties**

Li-Feng Cai*^1^, Jie-Ming Zhan^1^, Jie Liang^1^, Lei Yang^1^, Jie Yin*^2^

^1^ College of Environmental and Biological Engineering, Fujian Provincial Key Laboratory of Ecology-Toxicological Effects ＆ Control for Emerging Contaminants, Putian University, Putian, Fujian, 351100, P. R. China

^2^ Department of Materials Science, Fudan University, Shanghai 200433, China

**^*^** Li-Feng Cai. [89437499@qq.com](mailto:89437499@qq.com)

* Jie Yin. 20110300019@fudan.edu.cn

**Tab. S1** Pore structure parameters of HPC prepared from various carbonization conditions

| Samples | S_BET_  (m^2^·g^-1^) | S_mic_  (m^2^·g^-1^) | S_ext_  (m^2^·g^-1^) | V_mic_  (cm^3^·g^-1^) | V_ext_  (cm^3^·g^-1^) | V_total_  (cm^3^·g^-1^) |
| --- | --- | --- | --- | --- | --- | --- |
| 700 ℃ | 736 | 414 | 322 | 0.17 | 0.71 | 0.88 |
| 800 ℃ | 1030 | 824 | 206 | 0.32 | 0.57 | 0.89 |
| 900 ℃ | 1868 | 1522 | 346 | 0.61 | 0.96 | 1.57 |
| 1000 ℃ | 2388 | 1892 | 496 | 0.75 | 1.21 | 1.96 |
| 3 h | 1868 | 1522 | 346 | 0.61 | 0.96 | 1.57 |
| 6 h | 1935 | 1604 | 331 | 0.64 | 0.88 | 1.52 |
| 10 h | 2221 | 1716 | 505 | 0.69 | 1.02 | 1.71 |
| 2 ℃·min^-1^ | 1079 | 803 | 276 | 0.32 | 0.60 | 0.92 |
| 5 ℃·min^-1^ | 1868 | 1522 | 346 | 0.61 | 0.96 | 1.57 |
| 10 ℃·min^-1^ | 842 | 649 | 192 | 0.25 | 0.48 | 0.73 |


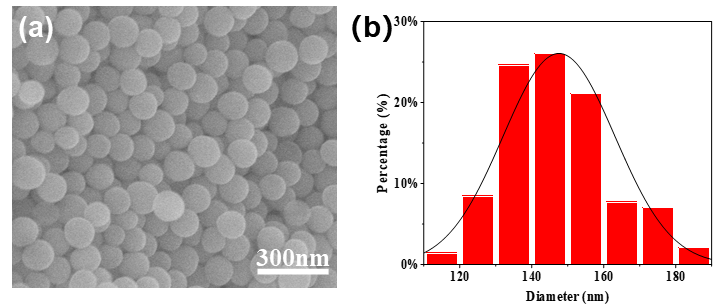


**Fig. S1** (a)SEM graphs and (b)Size distribution analysis of R-SiO_2_ nanoparticles

The SEM image of R-SiO_2_ nanoparticles was shown in Figure S1a. After grafting the silane, the appearance of R-SiO_2_ nanospheres still maintains a integrated spherical structure. The particle size distribution statistics (**Fig. S1b**) show that the average particle size of R-SiO_2_ nanospheres is about 147 nm, which is about 3 nm thicker than that of SiO_2_ nanospheres. It is because that the silane was grafted on the surface of the silica, wrapping the surface of the SiO_2_ nanospheres, and forming a thin grafted layer on the outside.

| 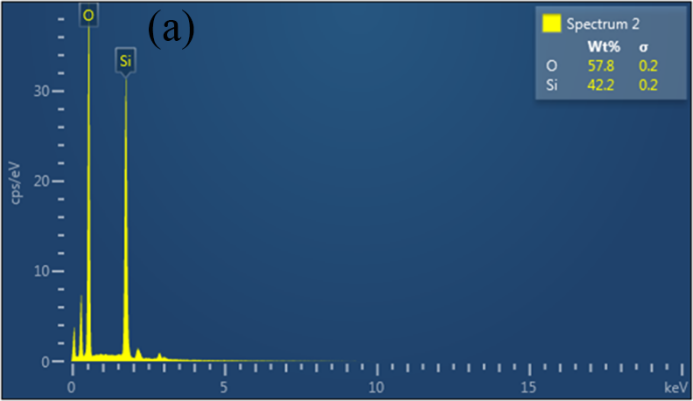 |
| --- |
| 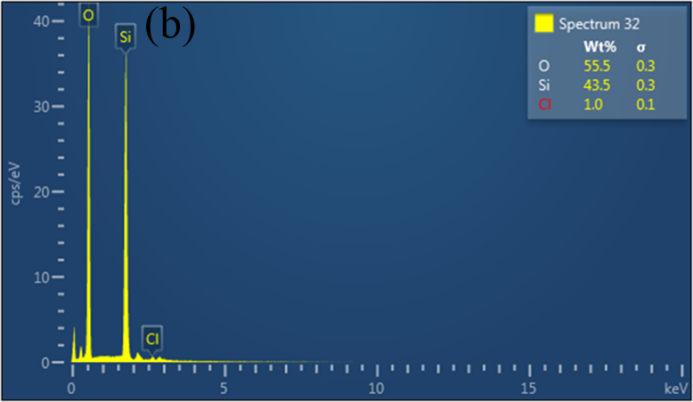 |

**Fig. S2** EDS graphs of (a)SiO_2_ nanoarticles and (b)R-SiO_2_ nanoarticles

The Energy Dispersive Spectrometer (EDS) analysis (**Fig. S3**) shows that the main elements of SiO_2_ nanospheres are silicon and oxygen. According to the analysis of the element content ratio in **Tab. S2**, the mass percentages of Si and O in SiO_2_ nanospheres are 42.2% and 57.8%, respectively, and the mass percentages of Si and O in R-SiO_2_ nanospheres are 43.5% and 55.5%, which the proportion of elements has almost no change. Excluding the interference of background elements, compared with the SiO_2_ nanospheres before modification, the R-SiO_2_ nanospheres showed a weak peak of Cl element at 2.53 keV in the low energy region. As the introduction of benzyl silane to the R-SiO_2_ during the modification process, the source of the Cl element was from the chloromethyl.

**Fig.S3** FT-IR spectrum of SiO_2_ and R-SiO_2_

**Tab. S2** Element content ratio of SiO_2_ nanoarticles and R-SiO_2_ nanoarticles

| Sample | O(Wt/%) | Si(Wt/%) | Cl(Wt/%) |
| --- | --- | --- | --- |
| SiO_2_ | 57.8 | 42.2 | - |
| R-SiO_2_ | 55.5 | 43.5 | 1.0 |

It can be seen from the infrared spectrum (**Fig. S3**) that both SiO_2_ and R-SiO_2_ have absorption peaks near 1065 cm^-1^and 801 cm^-1^, which belongs to the bending vibration peak of the Si-O-Si bond. The absorption peaks at 1408 cm^-1^ and 1607 cm^-1^ are derived from the vibration of the benzene ring skeleton, and the absorption peaks at 1507 cm^-1^, 1482 cm^-1^ and 1448 cm^-1^ are derived from the carbon-carbon double bond in the benzene ring. It indicate that the benzene ring structure is introduced into R-SiO_2_. In addition, the peak at 2896 cm^-1^ is derived from the stretching vibration of CH on the olefin. The above results further confirm that the reactive template R-SiO_2_ containing benzyl groups on the surface has been successfully prepared.

**Fig. S4** FT-IR spectrum of R-SiO_2_@xDCX

As shown in the infrared spectrum (**Fig. S4**), the peak at 651 cm^-1^ and 715 cm^-1^ was originated from the vibration of C-Cl and the stretching vibration of benzene ring, respectively. The absorption peak at 2930 cm^-1^ belongs to the vibration of CH in the methylene group, which proves that it forms a methylene crosslink bridge after the hypercrosslinking reaction. The information above indicate that, with the catalysis of FeCl_3_, the chloromethyl group on R-SiO_2_ was react with the benzene ring, forming a methylene cross-linked bridge between the template and the reactive monomer.

| 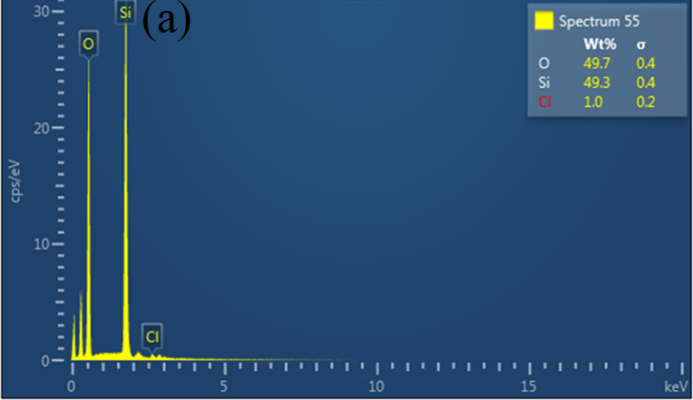 |
| --- |
| 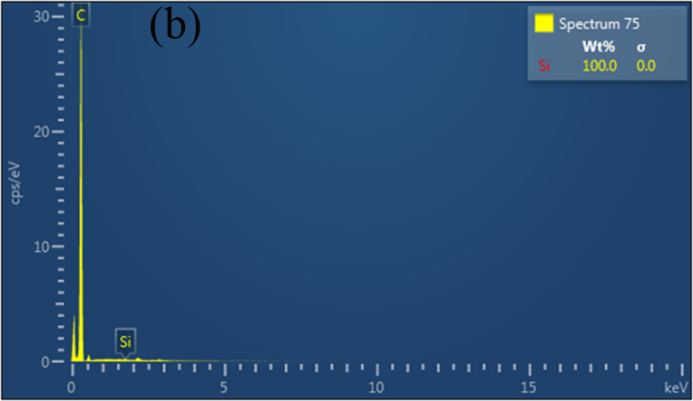 |

**Fig. S5** EDS graphs of (a) R-SiO_2_@xDCX nanoarticles before acid cleaning and

(b) R-SiO_2_@xDCX nanoarticles after acid cleaning (HPP)

The Energy Dispersive Spectrometer (EDS) analysis was presented in **Fig. S5**, which show a mass ratio of O element for R-SiO_2_@xDCX before HF etching is at 49.7 wt% (**Fig. S5a**), and is negligible after washing by HF (**Fig. S5b**), indicating SiO_2_ nanospheres were succesfully removed. After removing the templetes, the HPP nanospheres still contain a large amount of C element (**Fig. S5b**). It is because that the methylene crosslinking bridges are formed during the hypercrosslinking reaction, which constitutes the interconnected skeleton of the hollow nanospheres.

**Tab. S3** Element content ratio of R-SiO_2_@xDCX nanoarticles and HPP nanoarticles

| Sample | O(Wt%) | Si(Wt%)) | Cl(Wt%) |
| --- | --- | --- | --- |
| R-SiO_2_@xDCX | 49.7 | 49.3 | - |
| HPP | - | - | - |

**Fig. S6** TG curves of SiO_2_, R-SiO_2_ and R-SiO_2_@DCX nanoarticles

The thermogravimetric analysis results (**Fig. S6**) show that the sample has been through three stages in the thermogravimetric process. In the first stage, when the temperature is less than 100 ℃, the weight of the R-SiO_2_@xDCX sample drops rapidly, which may be due to the volatilization of the moisture inside the sample. When the temperature rises from 150 ℃ to 500 ℃, the rate of decrease of the sample weight remains stable, as the surface grafted polymer was losing The following stage, which was from 500 ℃ to 600 ℃, excpet for the SiO_2_ nanospheres, the R-SiO_2_ and the R-SiO_2_@xDCX nanospheres are both losing weight. In addition, the sample of R-SiO_2_@xDCX lose more weight than the R-SiO_2_, as the existence of the cross-linked polymer composed of chloromethyl on the surface, indicating the occurrence of cross-linking reaction. When the temperature reaches to the 800 ℃，the weight loss rate of the SiO_2_ nanospheres reaches 7.3%, which may cause by the lose of functional group on the SiO_2_ surface. In comparation, the weight loss rate of R-SiO_2_ nanospheres is 18.5%, which is 11.2 % more than the sample of SiO_2_, indicating that benzyl silane is successfully grafted to the surface of SiO_2_ nanoparticles. The weight loss rate of R-SiO_2_@xDCX nanospheres is 46% at 800 ℃, demonstrating that the nanospheres have been completely wrapped by the cross-linked polymer after the super-crosslinking reaction.

**Fig. S7** XRD graphs of SiO_2_, R-SiO_2_ and R-SiO_2_@xDCX nanoarticles

The XRD diffraction pattern (**Fig. S7**) shows that the SiO_2_ nanosphere has a weak peak at 2θ=23°, which is a characteristic peak of silica. After the super-crosslinking reaction, the intensity of the peak at 2θ=16°gradually increases, combined with hafl-width of the peak becomes wider. The fixation of the characteristic peak indicates the reaction from SiO_2_ to R-SiO_2_ to R-SiO_2_@xDCX nanospheres, amorphous structures maintain.

**Tab. S4** Summary of the maximum adsorption capacity of MB on various carbon adsorbents

| **Sample** | **S_BET_**  **(m^2^g^-1^)** | **Maximum adsorption capacity (mg·g^-1^)** | **References** |
| --- | --- | --- | --- |
| **HPC** | **2388** | **658.2** | **This work** |
| Activated carbon | 426–870 | 70.1–96.9 | 1 |
| Activated carbon foams | 2172 | 592 | 2 |
| Mesoporous activated carbon fibers | 1688-1825 | 367.5-490.9 | 3 |
| Hierarchical porous carbons | 1004 | 230 | 4 |
| Activated Carbons | 1,445 | 438 | 5 |
| Porous Biomass Charcoal | 554 | 24 | 6 |

**Reference**

1. Mopoung, S., Dejang, N. Activated carbon preparation from eucalyptus wood chips using continuous carbonization–steam activation process in a batch intermittent rotary kiln. Sci Rep 11, 13948 (2021).

2. Udayakumar M., El Mrabate B., Koós T., Szemmelveisz K., Kristály F., Leskó M., Filep Á., Géber R., Schabikowski M., Baumli P., Lakatos J., Tóth P., Németh Z. Synthesis of activated carbon foams with high specific surface area using polyurethane elastomer templates for effective removal of methylene blue. *Arab. J Chem.* **14**, 103214 (2021).

3. [Liu B.,](https://scholar.cnki.net/home/search?sw=6&sw-input=Liu%20B.) [Du C.,](https://scholar.cnki.net/home/search?sw=6&sw-input=Du%20C.) [Chen J. J.,](https://scholar.cnki.net/home/search?sw=6&sw-input=Chen%20J.J.) [Zhai J. Y.,](https://scholar.cnki.net/home/search?sw=6&sw-input=Zhai%20J.Y.) [Wang Y.,](https://scholar.cnki.net/home/search?sw=6&sw-input=Wang%20Y.) [Li H. L.](https://scholar.cnki.net/home/search?sw=6&sw-input=Li%20H.L.) Preparation of well-developed mesoporous activated carbon fibers from plant pulp fibers and its adsorption of methylene blue from solution. [*Chem Phys Lett*](https://scholar.cnki.net/journal/index/SJES000926140073)*.* **771**, 138535 (2021).

4. [Feng Z. Y.,](https://scholar.cnki.net/home/search?sw=6&sw-input=Zhi-Yuan%20Feng) [Meng](https://scholar.cnki.net/home/search?sw=6&sw-input=Long-Yue%20Meng) L. Y. Hierarchical porous carbons derived from corncob: study on adsorption mechanism for gas and wastewater. [*Carbon Lett*](https://scholar.cnki.net/journal/index/SSJD223349983526)*.* **31**, 643-653 (2021).

5. Zubir, M.H.M., Zaini, M.A.A. Twigs-derived activated carbons via H_3_PO_4_/ZnCl_2_ composite activation for methylene blue and congo red dyes removal. *Sci Rep* **10**, 14050 (2020).

6. Bai, S., Wang, T., Tian, Z. et al. Facile preparation of porous biomass charcoal from peanut shell as adsorbent. *Sci Rep* **10**, 15845 (2020).
